# Innovative insights into extrachromosomal circular DNAs in gynecologic tumors and reproduction

**DOI:** 10.1093/procel/pwad032

**Published:** 2023-05-26

**Authors:** Ning Wu, Ling Wei, Zhipeng Zhu, Qiang Liu, Kailong Li, Fengbiao Mao, Jie Qiao, Xiaolu Zhao

**Affiliations:** State Key Laboratory of Female Fertility Promotion, Center for Reproductive Medicine, Department of Obstetrics and Gynecology, Center for Reproductive Medicine, Peking University Third Hospital, Beijing 100191, China; National Clinical Research Center for Obstetrics and Gynecology, Peking University Third Hospital, Beijing 100191, China; Key Laboratory of Assisted Reproduction (Peking University), Ministry of Education, Beijing 100191, China; Beijing Key Laboratory of Reproductive Endocrinology and Assisted Reproductive Technology, Beijing 100191, China; Institute of Medical Innovation and Research, Peking University Third Hospital, Beijing 100191, China; Cancer Center, Peking University Third Hospital, Beijing 100191, China; Institute of Medical Innovation and Research, Peking University Third Hospital, Beijing 100191, China; Cancer Center, Peking University Third Hospital, Beijing 100191, China; State Key Laboratory of Female Fertility Promotion, Center for Reproductive Medicine, Department of Obstetrics and Gynecology, Center for Reproductive Medicine, Peking University Third Hospital, Beijing 100191, China; National Clinical Research Center for Obstetrics and Gynecology, Peking University Third Hospital, Beijing 100191, China; Key Laboratory of Assisted Reproduction (Peking University), Ministry of Education, Beijing 100191, China; Beijing Key Laboratory of Reproductive Endocrinology and Assisted Reproductive Technology, Beijing 100191, China; Department of Biochemistry and Biophysics, Beijing Key Laboratory of Protein Posttranslational Modifications and Cell Function, School of Basic Medical Sciences, Peking University Health Science Center, Beijing 100191, China; Institute of Medical Innovation and Research, Peking University Third Hospital, Beijing 100191, China; Cancer Center, Peking University Third Hospital, Beijing 100191, China; State Key Laboratory of Female Fertility Promotion, Center for Reproductive Medicine, Department of Obstetrics and Gynecology, Center for Reproductive Medicine, Peking University Third Hospital, Beijing 100191, China; National Clinical Research Center for Obstetrics and Gynecology, Peking University Third Hospital, Beijing 100191, China; Key Laboratory of Assisted Reproduction (Peking University), Ministry of Education, Beijing 100191, China; Beijing Key Laboratory of Reproductive Endocrinology and Assisted Reproductive Technology, Beijing 100191, China; Beijing Advanced Innovation Center for Genomics, Beijing 100191, China; Peking-Tsinghua Center for Life Sciences, Peking University, Beijing 100191, China; State Key Laboratory of Female Fertility Promotion, Center for Reproductive Medicine, Department of Obstetrics and Gynecology, Center for Reproductive Medicine, Peking University Third Hospital, Beijing 100191, China; National Clinical Research Center for Obstetrics and Gynecology, Peking University Third Hospital, Beijing 100191, China; Key Laboratory of Assisted Reproduction (Peking University), Ministry of Education, Beijing 100191, China; Beijing Key Laboratory of Reproductive Endocrinology and Assisted Reproductive Technology, Beijing 100191, China

**Keywords:** extrachromosomal circular DNAs, gynecologic tumors, reproduction, liquid biopsy, non-invasive prenatal testing

## Abstract

Originating but free from chromosomal DNA, extrachromosomal circular DNAs (eccDNAs) are organized in circular form and have long been found in unicellular and multicellular eukaryotes. Their biogenesis and function are poorly understood as they are characterized by sequence homology with linear DNA, for which few detection methods are available. Recent advances in high-throughput sequencing technologies have revealed that eccDNAs play crucial roles in tumor formation, evolution, and drug resistance as well as aging, genomic diversity, and other biological processes, bringing it back to the research hotspot. Several mechanisms of eccDNA formation have been proposed, including the breakage-fusion-bridge (BFB) and translocation–deletion–amplification models. Gynecologic tumors and disorders of embryonic and fetal development are major threats to human reproductive health. The roles of eccDNAs in these pathological processes have been partially elucidated since the first discovery of eccDNA in pig sperm and the double minutes in ovarian cancer ascites. The present review summarized the research history, biogenesis, and currently available detection and analytical methods for eccDNAs and clarified their functions in gynecologic tumors and reproduction. We also proposed the application of eccDNAs as drug targets and liquid biopsy markers for prenatal diagnosis and the early detection, prognosis, and treatment of gynecologic tumors. This review lays theoretical foundations for future investigations into the complex regulatory networks of eccDNAs in vital physiological and pathological processes.

## Introduction

Although the vast majority of eukaryotic cellular DNAs are packed in long linear chromosomes, other forms such as extrachromosomal circular DNAs (eccDNAs) have also long been found in various eukaryotes, including ciliates, drosophila, yeast, pigs, and humans (Hotta and Bassel, 1965; Hull et al., 2019; Paulsen et al., 2019; Molin et al., 2020; Wang et al., 2021a; Gao et al., 2023). Unlike previously identified circular mitochondrial, bacterial plasmid, and chloroplast DNAs, double-stranded or single-stranded circular eccDNAs occur in eukaryotic nuclei (Cohen and Lavi, 2009; Shibata et al., 2012; Cao et al., 2021; Zuo et al., 2021; Chen et al., 2022). EccDNAs are derived from but independent of chromosomal DNA, ranging in size from a few hundred to several million base pairs (bp) with most eccDNAs less than 1,000 bp (Ling et al., 2021). EccDNAs can also be classified as extrachromosomal DNA (ecDNA) or double minutes (DMs; 100 kb–several Mb), small polydispersed circular DNA (spcDNA; 100 bp–10 kb), microDNA (100–400 bp), or telomeric circles (t-circles; multiples of 738 bp) (Cohen et al., 1997; Regev et al., 1998; Tomaska et al., 2009; Storlazzi et al., 2010; Mehanna et al., 2017; Turner et al., 2017; Paulsen et al., 2019; Verhaak et al., 2019; Hung et al., 2021; Paulsen et al., 2021; Li et al., 2022; Noer et al., 2022). Their characteristics are shown and compared in Table 1. However, it should be noted that eccDNAs classification systems have varied among studies and researchers. Although eccDNAs were first discovered in wheat and pig germ cells in 1964, their functions have remained obscure because of their sequence homologous to linear DNA and the limitations of available detection methods. Nevertheless, the rapid development of sequencing technology and bioinformatics have accelerated the detection and characterization of small DNA fragments as well as eccDNAs, so the studies related to eccDNAs begin to enter the limelight frequently. Recent studies have shown that eccDNAs encode both regulatory elements (such as small RNAs) and full-length or truncated genes, playing multiple roles in a wide variety of organisms (Moller et al., 2018b; Paulsen et al., 2019; Shoshani et al., 2021; Bergstrom et al., 2022; Lin et al., 2022; Yi et al., 2022b).

**Table 1.** Classification of eccDNA.

eccDNA	Length	Characteristics	References
SpcDNA	100 bp–10 kb	spcDNAs mainly contain repetitive genome sequences. Elevated amounts of total spcDNAs are related to endogenous and induced genomic instability in rodent and human cells	Regev et al. (1998) and Noer et al. (2022)
MicroDNA	100 bp–400 bp	MicroDNAs are derived from unique non-repeating genomic regions, showing high gene density and high GC content. MicroDNAs are produced by DNA breaks associated with RNA metabolism or replication slip. They can amplify tRNA, microRNA, and Si-like RNA that can affect gene expression	Mehanna et al. (2017) and Paulsen et al. (2021)
EcDNA	100 kb–several Mb	EcDNAs (including DMs) are covalently closed, double-stranded, and lack centromere. EcDNAs contain multiple full genes and regulatory regions. EcDNAs are prevalent in human cancers and mediate high oncogene expression through gene amplification and regulation. EcDNAs are visible under a light microscope	Verhaak et al. (2019) and Hung et al. (2021)
Telomeric circles	Multiples of 738 bp	Telomeric circles are important for the immortalization of telomerase-negative cancers through the telomere lengthening (ALT) mechanism. Telomeric circles represent an evolutionarily conserved trait and may play important roles in telomere biology	Tomaska et al. (2009), Li et al. (2022) and Noer et al. (2022)

Gynecologic tumors such as ovarian and cervical cancers pose serious threats to women’s health. Except for cervical cancer, there is no reliable way to diagnose gynecologic cancers early; therefore, it is vital to elucidate the mechanisms associated with the occurrence of gynecologic tumors and develop sensitive biomarkers for their early diagnosis. The relationships between eccDNAs and gynecologic and other tumors have recently been investigated (Sun et al., 2015; Gu et al., 2020; Kim et al., 2020; Shoshani et al., 2021; Wang et al., 2021d; Cen et al., 2022). Firstly, eccDNAs mediate oncogene over-expression by amplifying more copies of oncogenes, carrying oncogene with more accessible chromatin, and increasing oncogene transcript levels as mobile super-enhancer elements (Yu et al., 2013; Turner et al., 2017; Kim et al., 2020; Zhu et al., 2021). These effects result in poor outcome for cancer patients. Secondly, eccDNAs contribute to drug resistance of cancer cells by carrying the drug resistance gene, or by using the reversible loss of the gain-of-function mutation of the drug target gene carried by eccDNAs (Pauletti et al., 1990; Raymond et al., 2001; Nathanson et al., 2014; Sun et al., 2015; Gu et al., 2020). Thirdly, eccDNAs may also promote heterogeneity and accelerate genomic evolution in tumors (Nathanson et al., 2014; Andor et al., 2016; Turner et al., 2017; deCarvalho et al., 2018). EccDNAs can be asymmetrically delivered to progeny cells via amitosis. Thus, cancer cells with more eccDNAs acquire a growth advantage over others (Wang et al., 2021c; Henssen, 2022; Sin et al., 2022; Yi et al., 2022b). Reproductive diseases such as infertility, multiple miscarriages, early labor, and birth defects negatively impact the health of the parents and their offsprings. For this reason, prevention, early intervention, and efficacious therapeutic modalities are urgently required for the foregoing conditions and disorders. Although eccDNAs have been long demonstrated to exist in the sperm of mammals, their roles in the reproductive system and its associated diseases are unknown. Recent studies confirmed the existence of eccDNAs in human sperm as well as their transient and permanent effects on germline inheritance (Henriksen et al., 2022). Moreover, eccDNAs occur in the plasma of pregnant women and may play roles in reproductive disease occurrence and progression (Sin et al., 2020; Sin et al., 2021). On this basis, eccDNAs could be highly valuable in the early diagnosis, treatment, and prediction of gynecologic tumors and other reproductive diseases.

The present review summarized the discovery history, biogenesis, currently available detection and analytical methods for eccDNAs, and deeply discussed the biological functions of eccDNAs, with a focus on their roles in gynecologic tumors and reproduction. Moreover, this review also elucidated the application of eccDNAs in prenatal diagnosis, drug targeting and liquid biopsy biomarkers in the early detection, treatment, and prognosis of gynecologic tumors.

## Research history of eccDNAs in gynecologic tumors and reproduction

**Figure 1.**
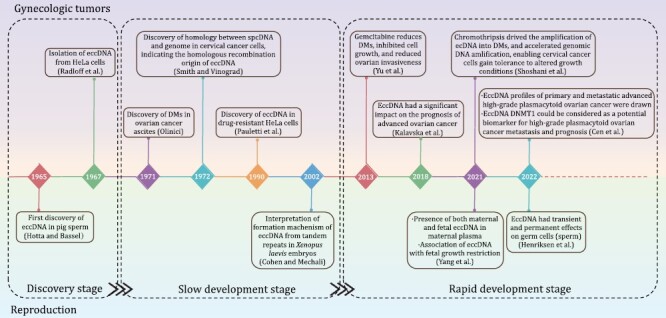
**Research history of eccDNAs in gynecologic tumors and reproduction.** Notable studies on eccDNAs in gynecologic oncology are listed on the upper side of the timeline. Milestone findings on eccDNAs in reproduction are shown below the timeline. The discovery, slow development, and rapid development research stages are separated by rectangular boxes.

For the present review, we divided the research history of eccDNAs in gynecologic tumors and reproduction into the discovery, slow development, and rapid development stages (Fig. 1). In the discovery stage, researchers focused mainly on eccDNA isolation and purification (Hotta and Bassel, 1965). EccDNAs with different sizes were first identified in pig sperm in 1965 (Hotta and Bassel, 1965) and in HeLa cells in 1967. Radloff et al. (1967) examined closed DNA from HeLa cytoplasm and detected closed-loop DNA in the size range of 0.2–2 µm. In the slow development stage, researchers focused mainly on the origins and classification of eccDNAs and began to explore their molecular functions. In 1971, double minute chromatin bodies were observed in ovarian carcinoma ascites cells at metaphase, ranging in size from G group chromosomes to double dots at the edge of visibility (Olinici, 1971). Besides, more detailed information had been added to the closed-loop DNA first found in HeLa cells since 1967: they were referred to as spcDNA and each cell contained 50–200 DNA circles (Radloff et al., 1967). Subsequent examinations of Chinese hamster ovary (CHO) cells revealed that: (i) they might exist both in nuclei and cytoplasm; (ii) most spcDNA were derived from repetitive sequences and were homologous to genomic DNA. Hence, repetitive sequences might frequently recombine and generate spcDNAs (Stanfield and Helinski, 1984). Other origins of eccDNA, such as non-homologous end linking and homologous recombination of tandem repeat sequences, were also proposed in later investigations (van Loon et al., 1994; Cohen and Mechali, 2002). Moreover, eccDNA was also preliminarily found to play a role in drug resistance in HeLa cells (Cohen and Mechali, 2002; Yu et al., 2013). During the rapid development stage, advances in high-throughput sequencing have helped clarify the roles of eccDNAs in gynecologic tumors and reproduction (Zhao et al., 2006). More connections between eccDNA and gynecologic tumors were found: (i) EccDNAs were targets of the cancer drug gemcitabine and were positively associated with ERK1/2 activation. The latter was a key component of the mitogen-activated protein kinase (MAPK) signaling pathway and was consistently dysregulated in malignant ovarian tumors (Yu et al., 2013; Sun et al., 2015); (ii) Dihydrofolate reductase (DHFR)-containing eccDNAs were detected in drug-resistant cervical cancer cells (Pauletti et al., 1990; Singer et al., 2000); (iii) eccDNAs significantly influenced the prognosis of advanced ovarian cancer. EccDNA DNMT1^circle10302690–10302961^ was a potential prognostic biomarker or therapeutic target of high-grade plasmacytic ovarian cancer metastasis (Kalavska et al., 2018; Cen et al., 2022); (iv) Chromothripsis was implicated in cancers and drove eccDNA-containing gene amplification in HeLa S3 cells (Shoshani et al., 2021). Besides, the value of eccDNAs in reproductive diseases and prenatal testing became manifestly evident during the rapid development stage. Researchers revealed that both maternal and fetal eccDNAs were present in maternal plasma, and the differences between them were investigated. EccDNA was also found in human sperm, and might have transient or permanent effects on germline inheritance. EccDNA was further detected in the urine of both healthy people and patients with chronic kidney disease (CKD), suggesting urine-derived eccDNA as a valuable non-invasive biomarker for diagnosis and monitoring of reproductive diseases (Lv et al., 2022). In addition, it was found that fetal growth restriction (FGR) might be linked to eccDNAs (Yang et al., 2021).

## EccDNA biogenesis

**Figure 2.**
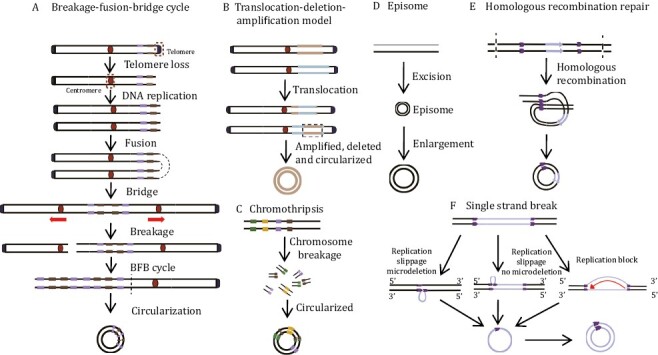
**EccDNA biogenesis models.** (A) Breakage-fusion-bridge (BFB) model. One chromosome loses its telomere and replicate to form two sister monotelomere-deficient chromatids that fuse at the non-telomere end. The double centromere-bridging chromosome product is then pulled apart to generate two unequal chromosomes. Telomere-free bridges are elongated by repetitive replication cycles and these repetitive sequences are eventually looped out as eccDNAs. (B) Translocation-deletion-amplification model. When translocation occurs, fragments adjacent to the translocation breakpoint might be amplified, deleted, and cyclized, resulting in eccDNAs. (C) Chromothripsis model. This process produces multiple small DNA fragments and some of these are linked and looped to form eccDNAs via DNA repair mechanisms. (D) Episome model. DNA fragments are excised from chromosomes and amplified by mutual recombination loops to produce eccDNAs. (E) Homologous recombination repair. Double-strand breaks occur in duplicated regions, and homologous recombination leads to the synthesis of eccDNA. (F) Single strand break. Replication slippage generates a loop on the template or product strand, which can both be excised and ligated into a loop, leaving either a microdeletion (left panel) or no microdeletion (center panel) on the chromosome. Replication fork stalls allow newly synthesized nascent DNA strands to circulate with the help of short microhomology stretches on the template (right panel). Single-strand circular DNA might then be converted by DNA polymerase into double-strand eccDNA.

EccDNA biogenesis varies with the environment (Fig. 2). In general, DNA replication, recombination, and damage repair are implicated in eccDNA production (van Loon et al., 1994; Singer et al., 2000; Vogt et al., 2004; Cohen et al., 2006). Although the details of eccDNA generation remain unclear, several models have been proposed, including the breakage-fusion-bridge (BFB) model, translocation–deletion–amplification model, chromothripsis model, and episome model (Stanfield and Lengyel, 1979; Stanfield and Helinski, 1984; Cohen and Mechali, 2001; Stephens et al., 2011; Ling et al., 2021). In addition, recent studies have also revealed that eccDNA production is closely associated with apoptosis (Wang et al., 2021d).

### Breakage-fusion-bridge model

The BFB model was proposed by McClintock and is the classic eccDNA formation model (Fig. 2A). The BFB cycle starts with the separation of the telomere from the chromosome (Murnane, 2012). If this double-strand break is not been repaired before DNA replication, the chromosome replicates during cell division and forms two sister monotelomere-deficient chromatids. The latter then fuses to form a double centromere-bridging chromosome. Under the opposing forces on the centromeres, the double centromere-bridging chromosome randomly breaks at anaphase into two monotelomere-deficient chromatids. BFB is repeated every cell cycle, thereby creating reverse repetitive sequences in one daughter cell and end deletions in the other. The unstable inverted repeats readily looped out and formed eccDNAs (Murnane and Sabatier, 2004).

### Translocation–deletion–amplification model

In the translocation–deletion–amplification model, DNA double-strand breaks occur and trigger reciprocal translocation (Fig. 2B). The DNA fragments near the translocation site can readily be amplified, excised, or deleted and circularized into eccDNAs (Van Roy et al., 2006). This model has been approved in two cases: (i) Van Roy et al. (2006) have demonstrated that nonsyntenic coamplification of proto-oncogene *MYC* and the AT motif binding factor 1 (*ATBF1*) was achieved through this model; (ii) Röijer et al. confirmed that the tumor-associated genes *HMGIC* and *MDMD2* in carcinoma ex pleomorphic adenoma co-amplified and overexpressed in the form of eccDNAs generated by this model.

### Chromothripsis model

Chromothripsis is a complex mutational process wherein numerous clustered chromosomal rearrangements occur in a single catastrophic event such as severe DNA damage along the length of a chromosome or chromosome segment (Fig. 2C). Chromothripsis occurs mainly in certain cancers and congenital diseases. Although the detailed mechanism of chromothripsis has not yet been elucidated, evidence shows that chromothripsis might cause eccDNA formation and evolution as follows: (i) Chromothripsis promotes eccDNA amplification in a manner dependent upon poly (ADP-ribose) polymerases (PARP) and the catalytic subunit of DNA-dependent protein kinase (DNA-PK) (Shoshani et al., 2021); (ii) structural evolution of eccDNAs might be achieved through tandem rounds of chromothripsis and lead to drug resistance in HeLa cells (Shoshani et al., 2021); (iii) recent studies have shown that consecutive BFB rounds can result in chromothripsis and, by extension, eccDNA generation (Umbreit et al., 2020); (iv) small eccDNAs can be closely associated with the late apoptosis that is included in chromothripsis, and Lig3 plays a role in the generation of cyclic nucleosome-sized DNAs. However, it remains to be determined whether large DMs and ecDNAs are also generated by chromothripsis (Wang et al., 2021d).

### Episome model

The episome model, also known as the “deletion-plus-episome” model, is another classic model of eccDNA formation. EccDNAs might be formed from a submicroscopic precursor called an episome which is derived from the chromosomal locus and replicated as an autonomous progenitor. In brief, DNA fragments are excised from chromosomes and either amplified by mutual recombination loops to form eccDNAs or integrated into chromosomes to form homologously stained regions (HSRs) (Graux et al., 2004) (Fig. 2D). This model has been proved in several cases: (i) *MYC*-containing eccDNAs in leukemia cases arose from excision and amplification (Storlazzi et al., 2010); (ii) progressively expanding DMs accompanied by deletions were detected in CHO cells (Carroll et al., 1988); (iii) the *EGFRvIII* harboring eccDNAs was excised from chromosomal DNA as episomes and circularized into eccDNAs, which has been well deciphered by the CRISPR-CATCH method developed by Howard Chang (Hung et al., 2022).

### Other mechanisms

EccDNAs are associated with chromosomal DNA damage and dysregulation. They might also be generated by DNA repair pathways such as interchromatid or interchromosome recombination between nonallelic homologs and microhomology-mediated DNA repair processes at double-strand DNA breaks (Vogt et al., 2004; Shibata et al., 2012) (Fig. 2E). Moreover, replication slippage (Fig. 2F) (Dillon et al., 2015) and chromosome recombination through tandem repeats might also produce circular DNAs (Cohen and Segal, 2009).

Genetic engineering tools have been employed to generate eccDNA *in vitro*. CRISPR-C was developed in 2019 and used to investigate eccDNA biogenesis, persistence, and cellular impact (Moller et al., 2018a). The core technology subtending CRISPR-C was the CRISPR/Cas9 guide RNA-mediated dual fluorescent biosensor cassette which visualizes specific fluorophore-expressing eccDNAs. CRISPR-C revealed that some eccDNAs are formed by end-joining-mediated DNA repair. Another study showed that ligase-assisted mini-circle accumulation (LAMA) created synthetic microDNAs mimicking known microDNA sequences (Paulsen et al., 2019). LAMA cyclically performed annealing, ligation, and denaturation to generate small circular DNAs. Using this method, researchers found that the artificial microDNAs expressed both functional small regulatory microRNAs (miRNAs) and novel si-like RNAs. The above engineering tools help researchers better understand the role of endogenous eccDNAs in different biological processes.

## Current eccDNA detection methods

### EccDNA imaging techniques

**Figure 3.**
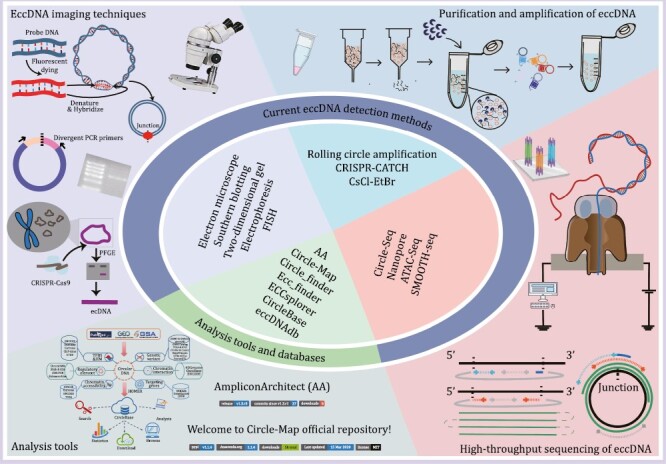
**Currently available tools for eccDNA detection and analysis.** Representative eccDNA imaging techniques, purification and amplification methods, high-throughput sequencing methods, and analytical tools are shown.

Before the development of sequencing technologies, researchers used light microscopy, electron microscopy (EM), fluorescence *in situ* hybridization (FISH), neutral two-dimensional (2D) gels, and other visualization methods to examine eccDNAs. Light microscopy could visualize large DMs but not small eccDNAs (Cox et al., 1965). Recent microscopy methods have evolved to allow the observation of fluorescently labeled eccDNA with structural illumination microscopy and the observation of eccDNA in its original form with scanning atomic force microscopy (Wang et al., 2021d; Henriksen et al., 2022). Moreover, Wu et al. (2019) combined super-resolution 3D structural illumination microscopy (3D-SIM) with scanning and transmission EM to confirm the circular structure of eccDNAs. FISH localized the eccDNA ring structure in the cells and distinguished extrachromosomally amplified oncogenes from those that were amplified on linear chromosomal DNA (Hung et al., 2021; Bergstrom et al., 2022). Since covalent closures and nicked loops delayed migration to a greater extent than linear molecules, 2D gels could characterize the circular eccDNA structure (Cohen and Lavi, 1996). ATAC-see identified the imaged components through direct imaging of the accessible genome *in situ*, cell sorting, and deep sequencing (Chen et al., 2016). It has been used to visualize eccDNA accessibility in mid-stage chromatin (Wu et al., 2019). In addition, EcSeg has also been developed to quantify DAPI (4ʹ,6-diamidino-2-phenylindole)-stained mid-term eccDNAs and showed how and where eccDNAs were repositioned as HSRs (Rajkumar et al., 2019) (Fig. 3).

### EccDNA purification and amplification

As the natural abundance of eccDNAs is low, they must be enriched for research purposes (Hotta and Bassel, 1965). CsCl-ethidium bromide (CsCl-EtBr) density gradient centrifugation was first used in 1967 to isolate closed eccDNA, and later became the standard method to isolate eccDNAs (Radloff et al., 1967). However, it required large amounts of eccDNAs for effective detection and large amounts of eccDNAs were lost through co-migration with linear DNA in CsCl-EtBr density gradient centrifugation (Cohen and Lavi, 1996). In 2012, Shibata et al. (2012) purified eccDNAs by lysing nuclei in alkaline solutions, depleting the linear DNA with exonucleases, and concentrating the eccDNAs via rolling circular amplification (RCA). In 2017, Shoura et al. developed a cyclosome method combining CsCl-EtBr gradient centrifugation and linear DNA cleavage with exonuclease V. RCA was avoided via Tn5 transposition-based fragmentation and a tagging system directly targeting circular DNA. In this way, a bioinformatic signature identifying complete eccDNA assembly was developed (Shoura et al., 2017). In addition, the CRISPR-CATCH technique combined *in vitro* CRISPR-Cas9 treatment with pulsed field gel electrophoresis of agarose-entrapped genomic DNA to selectively purify megabase-sized eccDNAs at base-pair resolution (Hung et al., 2022) (Fig. 3). Other studies further combined high-throughput sequencing with protocols aimed at enriching eccDNAs (Shibata et al., 2012; Moller et al., 2015; Kumar et al., 2017; Shoura et al., 2017; Wang et al., 2023).

### High-throughput sequencing of eccDNA

Progress in high-throughput sequencing technology has facilitated eccDNA research. Taking advantage of its unique reads, including split reads and discordant paired-end reads, eccDNA could be distinguished from linear DNA using high-throughput sequencing (e.g., SMOOTH-seq data) (Fan et al., 2021). The copy number of eccDNA increased in whole genome sequencing (WGS) data as cellular amplification, and this approach is more suitable for detecting eccDNA with higher copy numbers. To overcome this shortage, Circle-seq, which combined the eccDNA enrichment with high-throughput sequencing, was developed (Moller, 2020) and optimized to catch these sparse or novel eccDNAs (Wang et al., 2021d). It is a sensitive, genome-scale technique to detect and enrich eccDNAs built on well-established bacterial plasmid purification and sequencing technologies (Moller et al., 2015). Briefly, eccDNAs were lysed in alkaline solutions and collected in an elution buffer by gravity flow-through in an ion exchange column. Then, extra linear chromosomes were digested by Plasmid-Safe DNase, after which, the remaining eccDNAs were further enriched by φ29 rolling circle amplification. Finally, high-throughput sequencing and custom eccDNA mapping software were used to identify the eccDNAs. Recently, nanopore sequencing has been used to interpret full-length eccDNA sequences, since long-read repeated sequences arising from a single type of eccDNA could generate consensus sequences that match it. Thus, it can reveal the origin, biogenesis, and immunostimulatory function of eccDNAs (Wang et al., 2021d). Study has also shown that it can detect ~10^4^ eccDNAs at nucleotide resolution in ~10^6^ human myocyte nuclei (Moller, 2020) (Fig. 3). Moreover, other methods, including ChIP-seq, 4C-seq, PLAC-seq and ATAC-seq, have combined eccDNA sequence characterization, chromosome accessibility and epigenetic regulation to examine eccDNA characteristics (Wu et al., 2019; Kumar et al., 2020).

## Current eccDNA analysis tools and databases

A range of bioinformatic tools and databases have been developed to identify and predict the potential functions of these eccDNAs, which vary according to the type of eccDNA sequencing data and the prediction purposes (Fig. 3).

Several tools are currently being used to analyze eccDNAs (Table 2). (i) AmpliconArchitect (AA; https://github.com/virajbdeshpande/AmpliconArchitect). It employs WGS data to reconstruct putative eccDNAs and other focal amplicon structures including BFB and heavily-rearranged and linear amplicons. AA has been extensively validated on multiple simulated and real datasets with wide coverage and high copy number (Deshpande et al., 2019). Later, AmpliconReconstructor (AR, https://github.com/jluebeck/AmpliconReconstructor) was adapted from AA and integrated optical mapping (OM) of long DNA fragments (>150 kb) with NGS to improve single-nucleotide eccDNA resolution (Luebeck et al., 2020). (ii) Circle-Map (https://github.com/iprada/Circle-Map). It detects eccDNAs based on a full probabilistic model for aligning reads across the breakpoint of each eccDNA (Prada-Luengo et al., 2019). Thus, Circle-Map increases circular DNA detection sensitivity and precision for both simulated and real data. (iii) Circle_finder (https://github.com/pk7zuva/Circle_finder). It identifies circular DNA from the paired-end data generated by ATAC-seq, WGS, and whole exome sequencing (Su et al., 2021). However, circular DNA must be purified before library preparation, since the Circle_finder algorithm can’t distinguish between an extrachromosomal circle and chromosomal segmental tandem duplication. (iv) Ecc_finder (https://github.com/njaupan/ecc_finder). It analyzes single-molecule real-time sequencing data, including those from Pacific Biosciences and Oxford Nanopore Technologies, to predict the presence of eccDNAs (Zhang et al., 2021). Moreover, Ecc_finder also performs *de novo* eccDNA analysis without a reference genome, thereby expanding the theoretical range of species that can be studied with eccDNAs. (v) ECCsplorer (https://github.com/crimBubble/ECCsplorer). It detects eccDNA candidates from eccDNA-enriched data (Mann et al., 2022). It comprises two procedures that can be run together or separately depending on data availability: the first identifies informative read distributions, including high coverage, discordant mapping, and split reads, by mapping reads to the reference genome; the second identifies specifically enriched DNA circles by making reference-free comparisons of amplified eccDNA reading clusters with control sample data.

**Table 2.** Current analysis tools for eccDNA.

Analysis tools	Suitable read length	Suitable for large genomes	Genomic reference needed	Duplicate sites considered	EccDNA enrichment needed	Input data	Website	References
AmpliconArchitect	Short	No	Yes	No	No	WGS	https://github.com/virajbdeshpande/AmpliconArchitect	Deshpande et al. (2019)
Circle-Map	Short	No	Yes	Yes	Yes	Circle-seq	https://github.com/iprada/Circle-Map	Prada-Luengo et al. (2019)
Circle_finder	Short	No	Yes	No	No	ATAC-seq, WGS	https://github.com/pk7zuva/Circle_finder	Kumar et al. (2017)
ecc_finder	Short and long	Yes	No	Yes	Yes	Circle-seq	https://github.com/njaupan/ecc_finder	Zhang et al. (2021)
ECCsplorer	Short	Yes	No	No	Yes	Circle-seq	https://github.com/crimBubble/ECCsplorer	Mann et al. (2022)

Recently developed databases, such as CircleBase (http://circlebase.maolab.org) (Zhao et al., 2022), eccDNAdb (http://www.eccdnadb.org) (Peng et al., 2022), and eccDNA Atlas (http://lcbb.swjtu.edu.cn/eccDNAatlas) (Zhong et al., 2023), integrated and annotated the putative roles of eccDNAs (Table 3). They provide user-friendly interfaces for searching, browsing, and analyzing distinct eccDNAs, screening potentially functional varieties, and clarifying their molecular mechanisms in human cancers and other diseases.

**Table 3.** Current database for eccDNA.

Database	Website	Number of eccDNA	Species	Function	References
CircleBase	http://circlebase.maolab.org	601,036	*Homo sapiens*	Scores eccDNAs and helps to interpret the potential functions of eccDNA in the human genome using a powerful system, combined with six annotation sections, including “targeting genes”, “epigenetic regulations”, “regulatory elements”, “chromatin accessibility”, “chromatin interactions”, and “genetic variants”	Zhao et al. (2022)
eccDNAdb	http://www.eccdnadb.org	1,270	*Homo sapiens*	Acquires known and novel eccDNAs in cancers by computational analysis of WGS data, and annotates and illustrates their potential role in human cancer	Peng et al. (2022)
eccDNA Atlas	http://lcbb.swjtu.edu.cn/eccDNAatlas	639,313	*Homo sapiens, Arabidopsis thaliana,* *Drosophila* *Mus musculus,* *Cricetulus griseus,* Yeast, *Gallus gallus*	Provides a high-quality and integrated resource for browsing, searching and analyzing eccDNAs, such as sequence, function/characteristic, validation strategies from multiple species	Zhong et al. (2023)

## Functions of eccDNAs in gynecologic tumors and reproduction

**Figure 4.**
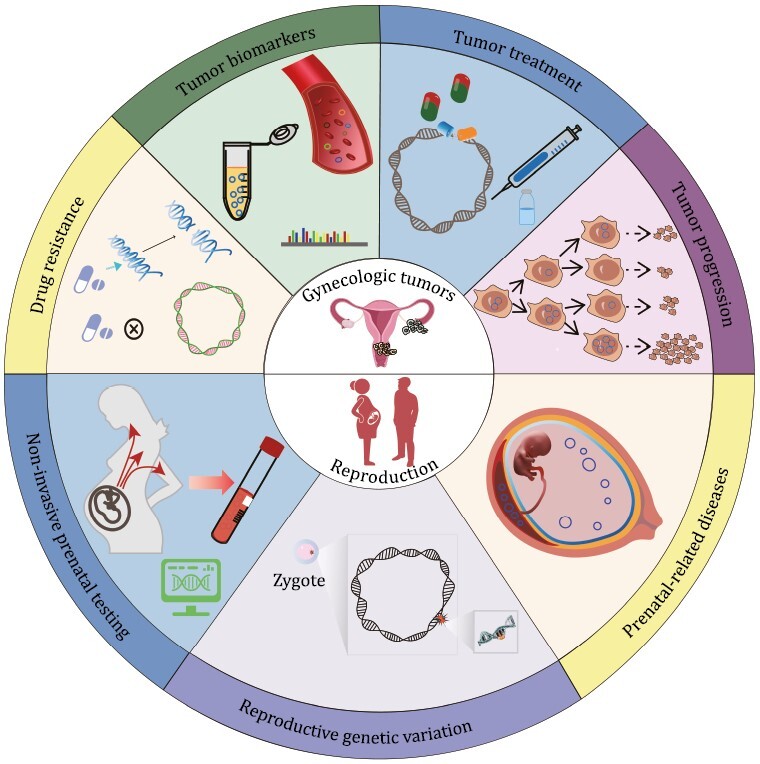
**EccDNA functions.** Recognized and predicted functions of eccDNA in gynecologic tumors and reproduction are shown in the upper and lower parts, respectively.

EccDNAs play important roles in cancer development, drug resistance, aging, genome diversity and other biological processes, and previous reviews mainly focused on their functions in cancer (Liao et al., 2020; Yan et al., 2020; Ling et al., 2021). Here, we summarized its function from a brand-new view of reproductive health, including its functions in gynecologic tumors and reproduction (Fig. 4).

### EccDNAs in gynecologic tumors

#### EccDNAs in gynecologic tumor progression

EccDNAs occurred in ovarian and cervical carcinomas and their related cell lines (Olinici, 1971; Atkin et al., 1983; McGill et al., 1993; Guan et al., 2001; Jin et al., 2012). EccDNAs mediated gynecologic oncogene over-expression by carrying more copies of oncogenes, carrying oncogenes with more accessible chromatin, and increasing oncogene transcript levels as mobile super-enhancer, which leads to tumor progression and poor outcome of gynecologic tumor patients (Guan et al., 2001; Yu et al., 2013; Turner et al., 2017; Kim et al., 2020; Ling et al., 2021). Researchers have demonstrated that oncogenes *PIK3CA, MCL1*, *MYCN*, *DHFR*, and *eIF-5A2* were over-amplified via eccDNA in primary ovarian cancers, ovarian cancer cell line UACC-1598 and cervical cancer cell line HeLa, respectively (Radloff et al., 1967; McGill et al., 1993; Guan et al., 2001; Sun et al., 2015). The high copy numbers of oncogene-containing eccDNAs resulted from the unequal segregation of eccDNA during cell division and environmental stress (Lange et al., 2022). Hence, eccDNAs provided growth advantages to cancer cells and promoted tumor progression (Yi et al., 2022a). Other study found novel matrix attachment regions (MARs) in a 682kb eccDNA in ovarian cancer cell line UACC-1598, which might be involved in eccDNA-mediated oncogene activation (Jin et al., 2012). Thus, eccDNAs might have mediated oncogene upregulation and activation here. Research on breast cancers has also shown that eccDNAs co-amplified the oncogene *ERBB2* and its associated enhancers, increased cell adaptability to the cancer environment, and promoted tumor progression (Morton et al., 2019).

#### EccDNAs as tumor biomarkers

Cell-free nucleic acid levels in the blood reflect pathological processes in the body such as benign and malignant lesions. Therefore, cell-free nucleic acids are convenient diagnostic biomarkers that do not necessitate tumor tissue biopsy (Schwarzenbach et al., 2011). Reliable early diagnosis is not feasible for any gynecologic malignancy except cervical cancer. For this reason, sensitive biomarkers for the early diagnosis of other gynecologic cancers are urgently required. A previous work detected tumor-derived human eccDNAs in the blood of a mouse xenograft model of human ovarian cancer and demonstrated that they could serve as disease biomarkers since they resisted exonuclease digestion (Kumar et al., 2017). Moreover, plasma collected before tumor resection contained longer eccDNA compared with plasma collected several weeks after surgery of ovarian cancer patients (Kumar et al., 2017). Study has also found several differentially expressed eccDNAs varieties between the same primary and metastatic high-grade serous ovarian cancer (HGSOC); the decrease of DNMT1^circle10302690–10302961^ was associated with poor prognosis in HGSOC patients, indicating that eccDNA DNMT1^circle10302690–10302961^ could be considered as a potential biomarker. Another study demonstrated that eccDNAs could non-invasively predict the response of ovarian cancer to chemotherapy (Kalavska et al., 2018). These preceding findings suggested that plasma eccDNAs could be used as diagnostic and prognostic biomarkers of ovarian cancer. Nevertheless, due to the low concentration of eccDNAs in plasma, limited enrichment methods and high heterogeneity of eccDNAs, the practical use of plasma eccDNAs as gynecologic tumor biomarkers is still challenging.

#### EccDNAs in tumor treatment

Certain drug treatments could decrease oncogenic eccDNAs. Hence, eccDNAs could be applied as therapeutic targets for gynecologic tumors (Yu et al., 2013). Early in 2001, researchers reported that noncytotoxic doses of hydroxyurea could lower the oncogene-containing eccDNA levels in the tumor cells of certain patients with advanced ovarian cancer, thereby improving the effectiveness of conventional chemotherapy. Hydroxyurea might preferentially capture the eccDNAs in micronuclei, which might contain chromatin degrading nucleases (Von Hoff et al., 1991; Von Hoff et al., 1992; Shimizu et al., 1994; Raymond et al., 2001). Later in 2013, study also revealed that the anticancer drug gemcitabine damaged the eccDNAs in UACC-1598 cells and the unrepaired eccDNAs were subsequently transported into the micronuclei and removed from the cancer cells. As a result, eccDNA-borne oncogenes such as *EIF5A2*, *MYCN*, and *MCL1* were then downregulated in the UACC-1598 cells. The observed decreases in UACC-1598 cell growth, colony formation, and invasion indicated that their malignancy was attenuated (Yu et al., 2013). Moreover, a recent study further disclosed that ERK1/2 inhibitors effectively ameliorate eccDNA-harboring ovarian cancers characterized by constitutive ERK1/2 phosphorylation (Sun et al., 2015). Inhibition of ERK1/2 activation in eccDNA-containing and ERK1/2 constitutively phosphorylated ovarian cancer cells markedly reduced eccDNA abundance and eccDNA-borne oncogene amplification and expression. In conclusion, eccDNA-targeting drugs are promising therapeutic strategies against gynecologic tumors and the elimination of additional extrachromosomal amplification oncogene capacity provides an additional strategy for studying the relationship between oncogene over-expression, cell differentiation, and associated signaling pathways. Nevertheless, the clinical application of therapeutic strategies for eccDNA-containing gynecologic tumors is currently impractical as the pharmacodynamic profiles and antitumor efficacies of these treatment modalities are inadequate. Therefore, novel approaches are required to reduce the frequency of eccDNA occurrence during cancer treatment.

#### EccDNAs and cancer drug resistance

Drug resistance is a major challenge in gynecologic tumor treatment and may lead to cancer recurrence and metastasis (Mansoori et al., 2017). Tumor evolution and the upregulation of drug-resistance genes are the main factors contributing to the therapeutic recalcitrance of gynecologic tumors and eccDNAs promote tumor evolution and drug-resistance gene expression (Kalavska et al., 2018). Several studies have shown that eccDNAs increase intratumor heterogeneity in cervical and ovarian cancers (Pauletti et al., 1990). This unequal segregation and the massive amplification of oncogenes on eccDNA made it easy for cancer cells to adapt to the environment in which they evolved. There was a significant correlation between the presence of eccDNAs and the drug resistance of the human cervical cancer cell lines, suggesting that eccDNAs played a key role in the phenotype of the tumor cells (Shimizu et al., 2001; Lange et al., 2022). Further studies have revealed that additional chromothripsis events caused eccDNAs to undergo continuous structural evolution, which could increase drug tolerance in tumors (Shoshani et al., 2021).

### EccDNAs in reproduction

#### EccDNAs in non-invasive prenatal testing

Non-invasive prenatal testing (NIPT) has been widely used to detect fetal chromosomal and genetic anomalies in pregnancy (Wright and Burton, 2009; Cernat et al., 2019). With more quantity and variety of fetal-derived DNA being detected, the diagnostic application of NIPT is expanding (Pan et al., 2020). Researchers from Dennis Lo’s team, who is the father of NIPT, first identified both maternal and fetal-derived eccDNAs in maternal plasma (Sin et al., 2020). The authors disclosed that (i) plasma eccDNAs were longer than their linear counterparts whereas fetal eccDNAs were shorter than those of maternal origin, (ii) the eccDNA junctions exhibited dual-repeat patterns and were, therefore, nucleosomal in origin, and (iii) the closed circular structure of eccDNAs endued them with higher exonuclease resistance compared with their linear counterparts. For these reasons, eccDNAs are promising as NIPT biomarkers. Later in 2021, Lo’s team further analyzed fetal-derived eccDNAs in maternal plasma (Sin et al., 2021) and found that (i) they were hypomethylated compared with maternal eccDNAs, (ii) their methylation densities were positively correlated with their sizes, and (iii) they were rapidly cleared from the maternal blood after delivery, which was similar to fetal linear DNA. The foregoing study also reported the epigenetic information and methylation status of the fetal-derived eccDNAs in maternal plasma. Thus, fetal eccDNAs are potential biomarkers for the early diagnosis of preeclampsia and other disorders characterized by dysregulated DNA methylation. Moreover, investigating the relationships among abnormalities in maternal plasma eccDNA profiles and various pregnancy-associated conditions could facilitate and improve clinical NIPT application.

#### EccDNAs and reproductive genetic variation

Reproductive genetic variation is caused mainly by germ cell (gamete) mutation and recombination. It may be transmitted from one generation to another and could, therefore, affect population dynamics and evolution (Sirugo et al., 2019). EccDNAs can loop out or insert into the genome, thereby causing genetic variation and destabilizing the genome (Wang et al., 2021b). Moreover, as eccDNAs have been detected in the germ cells and embryos of various species, they might play important roles in reproductive genetic variation (Cohen and Mechali, 2001, 2002; Mouakkad-Montoya et al., 2021; Henriksen et al., 2022). A study conducted in 2021 showed that eccDNAs were predominantly derived from segmental duplications that form hotspots for copy number variations, and might promote human genetic variation (Mouakkad-Montoya et al., 2021). Another investigation performed in 2022 examined eccDNAs in human sperm and demonstrated that (i) eccDNA formation was inversely correlated with the meiotic recombination rate, (ii) chromosomes bearing the most coding genes and Alu elements formed the least eccDNA, and (iii) eccDNAs persisted in the human germline by reinserting into chromosomes and creating genomic alterations that were passed on to the next generation along with the germ cells. Interestingly, researchers also detected eccDNA formation during the embryonic development of *Xenopus* in cell-free egg extracts (Cohen and Segal, 2009). EccDNAs in unfertilized eggs and during early development may introduce plasticity that could (i) balance population-level genome integrity, and (ii) cause genetic variation among individual embryos from the same parent (Cohen and Mechali, 2001). Recently, CRISPR-CATCH has been developed for targeted eccDNA enrichment, which promoted the study on eccDNA source, methylation modification, and the associations between enhancers and genes. This method provides convenience to elucidate the roles of eccDNAs in genetic variation (Hung et al., 2022).

#### EccDNAs and prenatal-related diseases

Prenatal-related diseases such as preeclampsia and FGR threaten the health of mothers and fetuses (Surico et al., 2019). Clarification of the pathogenic mechanisms of prenatal-related diseases could facilitate the clinical treatment of these disorders. A recent study (Yang et al., 2021) on FGR disclosed that (i) the amount of placental eccDNA was significantly higher in patients with FGR than it was in normal individuals, (ii) a complex network and biological interactions may exist between eccDNAs and ncRNAs, and (iii) eccDNAs may drive FGR via immune signaling pathways. Moreover, another study on the methylation of maternal plasma eccDNA also indicated that eccDNAs might play a role in preeclampsia (Sin et al., 2021). However, researches on the relationship between eccDNAs and prenatal-related diseases are at the very beginning, and the detailed mechanism still remains largely unknown. Intensive studies need to be carried out in this new field, which is full of promise and challenges.

## Conclusions and perspectives

Gynecologic tumors and developmental disorders are major threats to human health and reproduction (Schwartz et al., 2019; Yang et al., 2019). As a rising star, the function of eccDNA in gynecologic tumors and reproduction has gained great widespread interest since its first discovery in pig sperm and ovarian cancer ascites (Hotta and Bassel, 1965; Olinici, 1971). The present review summarized the research history, biogenesis, and currently available detection and analytical methods of eccDNAs. In addition, it also focused on the roles of eccDNA in gynecologic tumors and reproduction. However, we could also conclude from the above review that there are still many technical limitations in the eccDNA research field: (i) How to monitor eccDNA *in vivo*? (ii) How to synthesize large eccDNA *in vitro*? (iii) How to cleave the targeted eccDNAs in tumor cells? (iv) How to identify the epigenetic modifications of eccDNA? (v) How to investigate the molecular functions of endogenous eccDNAs carrying diverse genomic segments?

Nonetheless, eccDNAs offer great promise in clinical reproductive medicine. First, eccDNAs could serve as therapeutic targets for gynecologic tumors, and novel drugs could be designed to cleave eccDNA-harboring oncogenes. Excitingly, Boundless Bio (San Diego, CA, USA) is a pioneer in the development of ecDNA-directed therapies (ecDTx) that could substantially improve the prognosis of patients with aggressive gene-amplified cancers. Second, eccDNAs could be applied as liquid biopsy markers for the diagnosis and prognosis of gynecologic tumors and diseases. Third, eccDNA could be a good biomarker for NIPT with more fetal eccDNA information being revealed in the near future. The present review clarified the roles of eccDNAs in reproductive health and disease and might enable future researchers to elucidate the complex regulatory networks of eccDNAs in various physiological and pathological processes.

## Data Availability

The data are all available in the article.

## Abbreviations

2D gels: two-dimensional gels
3D-SIM: 3D structural illumination microscopy
AA: AmpliconArchitect
AR: AmpliconReconstructor
ATBF1: AT motif binding factor 1
BFB: breakage-fusion-bridge
CHO: Chinese hamster ovary
CKD: chronic kidney disease
CsCl-EtBr: CsCl-ethidium bromide
DAPI: 4ʹ,6-diamidino-2-phenylindole
DHFR: dihydrofolate reductase
DMs: double minutes
DNA-PK: DNA-dependent protein kinase
eccDNAs: extrachromosomal circular DNAs
ecDNA: extrachromosomal DNA
ecDTx: ecDNA-directed therapies
EM: electron microscopy
FGR: fetal growth restriction
FISH: fluorescence in situ hybridization
HGSOC: high-grade serous ovarian cancer
HSRs: homologously stained regions
LAMA: ligase-assisted mini-circle accumulation
MAPK: mitogen-activated protein kinase
MARs: matrix attachment regions
miRNAs: microRNAs
NIPT: non-invasive prenatal testing
OM: optical mapping
PARP: poly (ADP-ribose) polymerases
RCA: rolling circular amplification
spcDNA: small polydispersed circular DNA
t-circles: telomeric circles
WGS: whole genome sequencing
